# Sequence signatures involved in targeting the male-specific lethal complex to X-chromosomal genes in *Drosophila melanogaster*

**DOI:** 10.1186/1471-2164-13-97

**Published:** 2012-03-19

**Authors:** Philge Philip, Fredrik Pettersson, Per Stenberg

**Affiliations:** 1 Deptartment of Molecular Biology, Umeå University, 901 87 Umeå, Sweden; 2 Computational Life Science Cluster (CLiC), Umeå University, 901 87 Umeå, Sweden; 3 UmBio, 907 19 Umeå, Sweden

**Keywords:** Dosage compensation, Sequence signatures, *Drosophila*, Motif discovery, MSL-complex

## Abstract

**Background:**

In *Drosophila melanogaster*, the dosage-compensation system that equalizes X-linked gene expression between males and females, thereby assuring that an appropriate balance is maintained between the expression of genes on the X chromosome(s) and the autosomes, is at least partially mediated by the Male-Specific Lethal (MSL) complex. This complex binds to genes with a preference for exons on the male X chromosome with a 3' bias, and it targets most expressed genes on the X chromosome. However, a number of genes are expressed but not targeted by the complex. High affinity sites seem to be responsible for initial recruitment of the complex to the X chromosome, but the targeting to and within individual genes is poorly understood.

**Results:**

We have extensively examined X chromosome sequence variation within five types of gene features (promoters, 5' UTRs, coding sequences, introns, 3' UTRs) and intergenic sequences, and assessed its potential involvement in dosage compensation. Presented results show that: the X chromosome has a distinct sequence composition within its gene features; some of the detected variation correlates with genes targeted by the MSL-complex; the insulator protein BEAF-32 preferentially binds upstream of MSL-bound genes; BEAF-32 and MOF co-localizes in promoters; and that bound genes have a distinct sequence composition that shows a 3' bias within coding sequence.

**Conclusions:**

Although, many strongly bound genes are close to a high affinity site neither our promoter motif nor our coding sequence signatures show any correlation to HAS. Based on the results presented here, we believe that there are sequences in the promoters and coding sequences of targeted genes that have the potential to direct the secondary spreading of the MSL-complex to nearby genes.

## Background

*Drosophila melanogaster *males are heterogametic (XY), while females are homogametic (XX). The Y chromosome has gradually lost genes and degenerated, resulting in an increasingly aneuploid condition in males and the evolution of systems that compensate for between-sex differences in doses of genes located on X chromosomes [1-4]. The dosage-compensation system equalizes X-linked gene expression between males and females, thus maintaining an appropriate balance between the expression of genes on X chromosome(s) and the autosomes [5,6].

The amount of transcripts from the single X chromosome of male *Drosophila* individuals is boosted about two-fold relative to levels of each of the two in females, thereby roughly equalizing their overall X chromosome gene expression [7]. This dosage compensation is critical, and loss of required proteins leads to male-specific lethality [8,9]. These proteins include MSL-1 (male-specific lethal 1), MSL-2, MSL-3, MOF (males absent on the first) and MLE (maleless), which form an X chromosome-specific MSL complex, or dosage compensation complex (DCC), with two functionally redundant long non-coding RNAs: RNA on the X1 and *X*2; *roX1 *and *roX2*, respectively [10-14]. The selective activation of X chromosomal genes is at least partly due to the hyperacetylation of histone H4 lysine 16 (H4K16) by the histone acetyltransferase (HAT) - MOF, an integral subunit of the MSL complex [15,16].

The binding pattern of MSL proteins on the X chromosome has been identified in diverse cell lines, embryos and third instar larvae using various genome-wide techniques such as chromatin immunoprecipitation coupled with microarray technology (ChIP-on-chip) or deep sequencing (ChIP-seq) [17-22]. Transcript levels of genes in RNAi-mediated depletion backgrounds and *msl *gene mutants have also been examined in diverse cell lines, embryos, and larvae using hybridization of transcript populations to gene expression microarrays or Real-time PCR [20,23-25]. These studies have revealed that: the MSL complex preferentially binds to gene coding regions, particularly the 3' end of genes; the binding pattern does not dramatically change during different stages of development; and loss of MSL-complex functionality only reduces expression of X-linked genes to about 80% of wild type levels. In addition, results of a recent analysis indicate that the MSL complex mediates dosage compensation of X chromosomal genes by enhancing transcriptional elongation, in accordance with the observed 3' bias [26].

Two main models have been proposed to explain the distribution of MSL complexes along the X chromosome. One suggests that the complex initially targets a relatively small number of X chromosome-specific primary recruitment or chromosomal "entry" sites (CES) then "spreads" along the chromosome from these sites in cis [27,28]. The other postulates that large numbers of specific sites of varying affinities are present, based on data gathered from X chromosomal translocation studies [29,30].

*In situ *hybridization analyses of polytene chromosomes have shown that the *Drosophila *X chromosome is enriched in (dC - dA)_n_/(dG - dT)_n _sequences [31], and that in every *Drosophila* species examined to date dosage-compensated chromosomes have higher than average CA/TG, CT/AG and C/G frequencies [32]. Subsequent, computational whole-genome sequence analysis showed that throughout the *Drosophila *genus X chromosomes can be distinguished from other chromosomes by their A, T, C/A_n _and G/T_n _repeat sequences [33,34]. Recent MSL protein-binding region analyses have also detected X chromosomal enrichment of low complexity sequence elements, such as GA- and CA-based dinucleotide repeats and runs of adenines [19,22,29,35]. In addition, GA-rich or TC-rich motifs have been identified in high affinity binding sites (HAS) for MSL proteins on the X chromosome using genome-wide techniques [18,22]. A repetitive sequence motif [G(CG)N]_4 _was also recently discovered in low affinity sites targeted by MSL proteins [36]. However, although the enrichment of simple sequence elements has been detected on the X chromosome it is still unclear if primary DNA sequences are involved in the targeting of the MSL complex to and within individual genes.

Here we present an extensive analysis of X chromosome sequence variation, and its potential involvement in dosage compensation, in which we used multivariate modeling and previously published data to explore relationships between MSL complex distributions, transcription patterns and five gene features -- promoters, 5' UTRs, coding sequences (CDS), introns, 3' UTRs -- and intergenic sequences (hereafter also classed as gene features, for convenience). Our results show that: the X chromosome has a distinct sequence composition within all six types of features examined; some of this variation correlates with genes targeted by the MSL-complex; the insulator protein BEAF-32 binds preferentially upstream of MSL-bound genes; BEAF-32 and MOF co-localizes in promoters; and bound genes have a distinct sequence composition that shows a 3' bias within coding sequence.

## Results

### The *Drosophila melanogaster *X chromosome has distinct sequence signatures

In a previous analysis of sequence variation between the *Drosophila melanogaster *(*Dm*) chromosomes we found evidence of chromosome-distinguishing sequence words on the fourth and X chromosomes [34]. In the study presented here we focus on the X chromosome and whether any of its sequence variation can be related to the dosage compensation of this chromosome. We excluded the 4^th ^chromosome (since it is atypical in many respects) from all our analyses.

**Figure 1:**
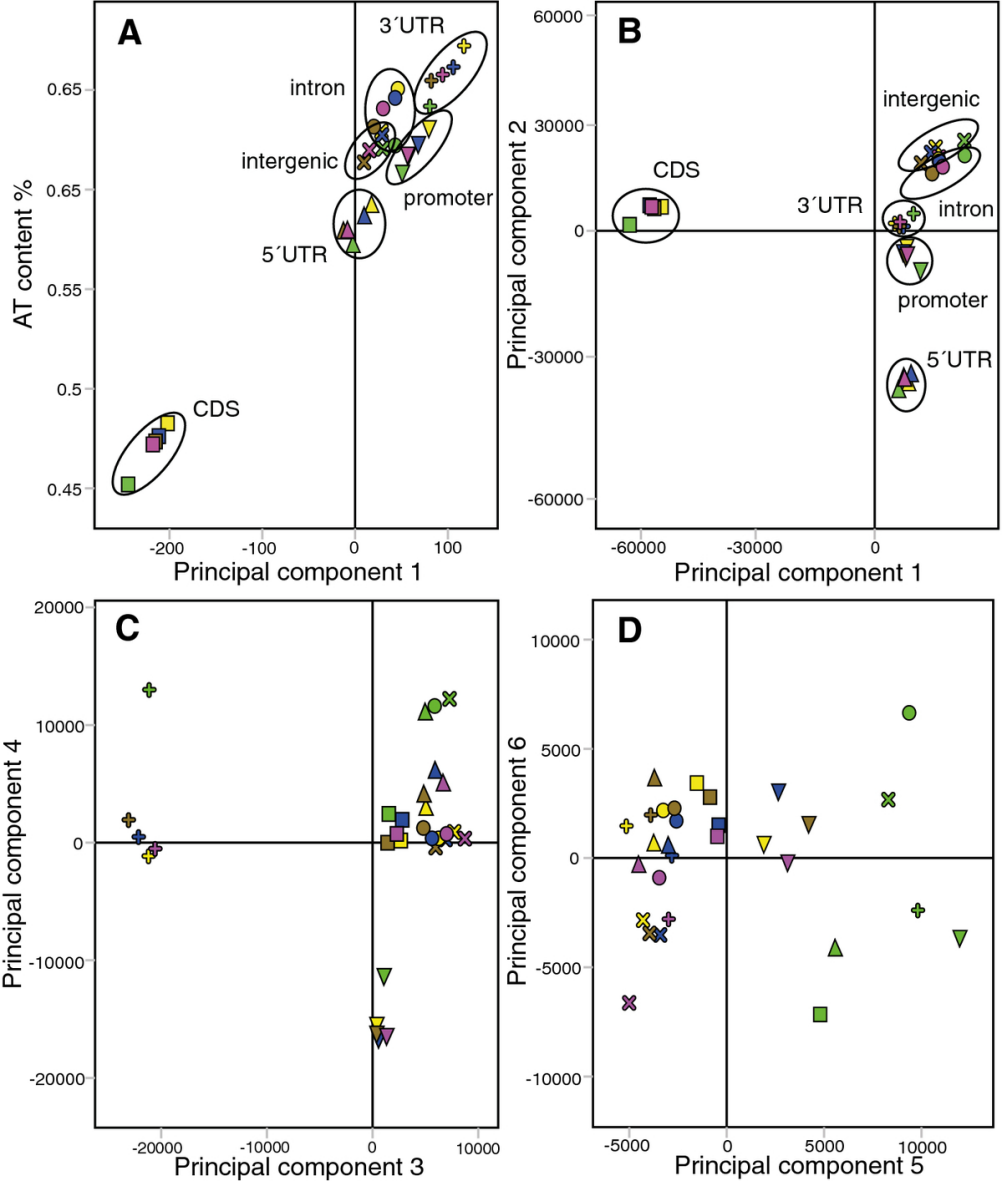
**PCA of *D. melanogaster *gene features**. Results of PCA of frequencies of sequence words within gene features in *Dm *chromosome arms. Chromosomes colour-coded, and gene features indicated by symbols as follows: green = X, magenta = 3R, brown = 2R, blue = 3L, yellow = 2L; ∇ = promoter, Δ = 5'UTR, □ = CDS, O = intron, + = 3'UTR, × = intergenic. (A) Scatter plot of PCA first component scores of the gene features versus their AT contents. (B), (C) and (D): 1^st ^vs 2^nd^, 3^rd ^vs 4^th ^and 5^th ^vs 6^th ^component score plots (R2cum = 0.774, 0.923 and 0.954, respectively) of the AT-normalized analysis of 2-6 mer frequencies in gene features.

To examine the sequence variation of the X chromosome systematically we divided the *Dm *genome into six sequence types (hereafter referred to as gene features): promoters (500 bp upstream of TSS), 5' UTRs, coding sequences, introns, 3' UTRs and intergenic sequences. Within these gene features we calculated the frequencies of all two to six base pair long sequence words and performed Principal Component Analysis (PCA) as in [34]. PCA summarizes the main variation in a multidimensional dataset, here consisting of 30 observations (the six gene features of the five major chromosome arms) and 5456 variables (all two to six base pair sequence words). As expected, the first Principal Component separates the observations based on AT-content (Figure 1A). In general the X chromosome has a lower AT-content than the autosomes (Additional file 1).

To study sequence variation that is not directly correlated to AT-content we applied the following simple normalization. The frequency of each sequence word was divided by its expected frequency in each observation, see [34] for details. Interestingly, the PCA based on the normalized values showed that there is more sequence variation between gene features than between chromosomes (Figure 1B). The first Principal Component separates coding sequences of all chromosomes from all other gene features and the second separates 5' UTRs. The other gene features separate in the proceeding components (Figure 1C). This has strong implications for attempts to identify discriminating patterns between groups of sequences; if the differences between gene features included are not accounted for, the results may reflect differences in gene feature composition rather than biologically relevant sequence variation.

In the fifth Principal Component of the gene feature PCA, all X-chromosomal gene feature sequences are shifted away from the autosomal sequences (Figure 1D; X-chromosomal sequences, shown in green, are shifted to the right). Corresponding loadings for the fifth component reveal that all gene features on the X chromosome are enriched in mono- and di-nucleotide repeats (Additional file 2; a summary of fits for all multivariate models is available in Additional file 3). The AT-normalized PCA models for each individual class of gene features also clearly showed that the X chromosome has a distinct sequence composition (Additional file 4), indicating that there are potential X chromosome targeting sequences within all of its gene features.

### Gene features of expressed genes strongly bound by MSL have distinct sequence signatures

We next explored whether any of the sequence signatures in the gene features correlated to binding of the Male-Specific Lethal (MSL) complex. For this purpose we used the data from [20] to select strongly MSL bound and weakly MSL bound genes as well as expressed and unexpressed genes (see Methods). We did not try to define genes that are bound or unbound by the MSL-complex, but rather select two extreme groups (one with very strong binding and one with very weak or no binding). The data from [20] currently represent the only dataset where mapping of several MSL-complex components and transcription in mutants/knock-downs of MSL-components are available in the same cell-type. Using several components of the MSL-complex should improve estimates of its binding values to genes, but we also compared the gene binding values obtained using the MOF, MSL-1 and MSL-3 data from [20] to gene binding values calculated in the same way based on data from [17,18,22,37] and modENCODE http://www.modencode.org. In general all data sets correlate very well despite being performed by different groups using different conditions and antibodies (average Spearman *R *= 0.83, Additional file 5). We conclude that estimates of MSL-binding values are consistent irrespective of which data sets are used.

**Figure 2:**
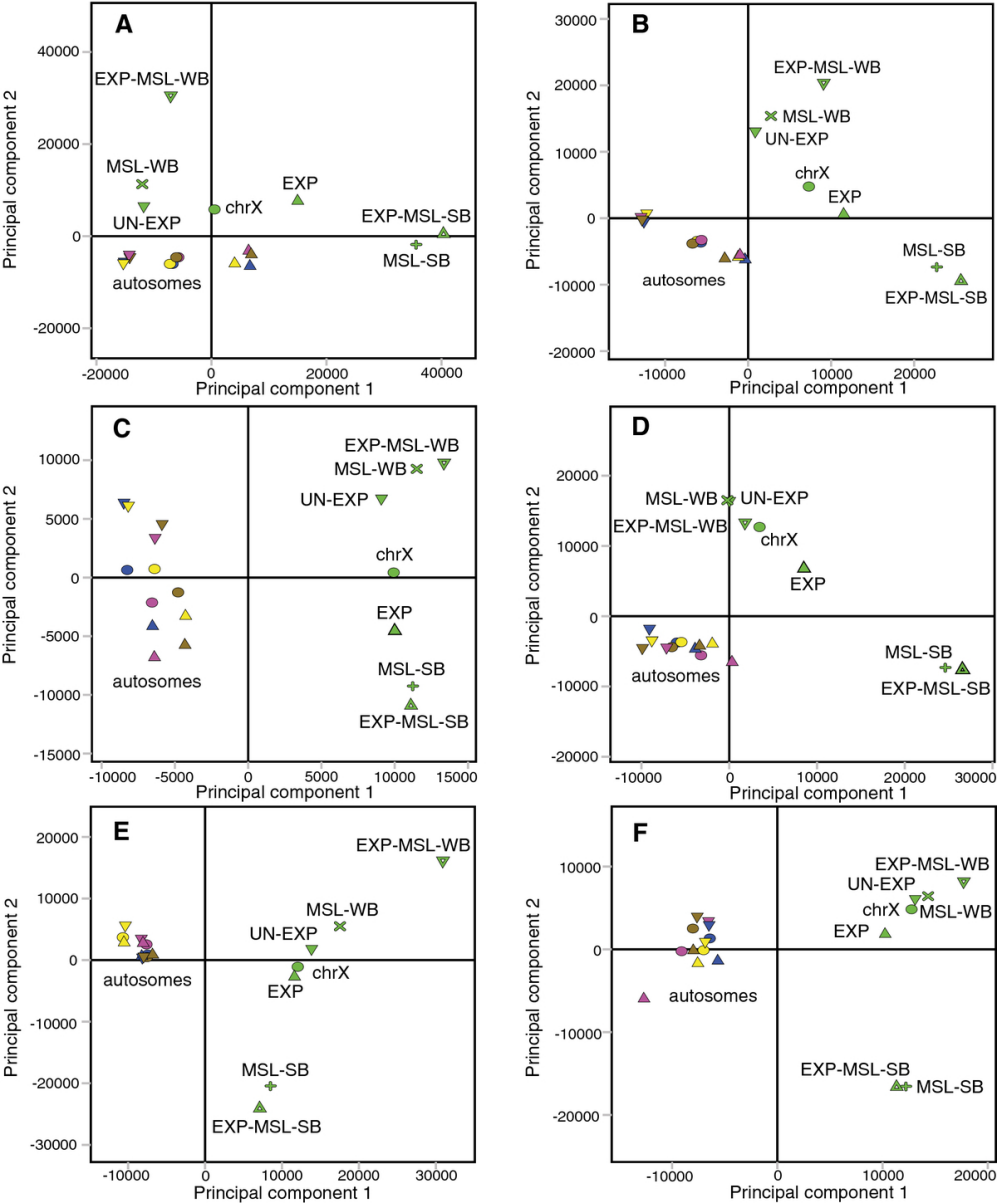
**PCA of gene features grouped by MSL binding and expression status**. Results of PCA of gene feature sequences of genes that are expressed/unexpressed and strongly/weakly bound by MSL. Chromosomes colour-coded, and gene features indicated by symbols as follows: green = X, magenta = 3R, brown = 2R, blue = 3L, yellow = 2L; O = all genes, Δ = expressed genes (EXP), ∇ = unexpressed genes (UN-EXP), + = MSL strongly bound genes (MSL-SB), × = MSL weakly bound genes (MSL-WB), Δ = expressed MSL strongly bound genes (EXP-MSL-SB), ∇ = expressed MSL weakly bound genes (EXP-MSL-WB). **(A)**, **(B)**, **(C)**, **(D)**, **(E) **and **(F) **show 1^st ^vs 2^nd ^component score plots (R2cum = 0.657, 0.517, 0.732, 0.872, 0.577 and 0.761, respectively) of the AT-normalized 2-6 mer promoter, 5' UTR, coding sequence, intron, 3' UTR and intergenic sequence analyses, respectively.

After AT-normalization (see Additional file 6 for AT-content differences) we again applied PCA to the data for each class of gene feature (separately), but this time incorporating information on MSL-binding and expression status (Figure 2). Interestingly, the first two Principal Components of each resulting PCA model not only separated X-chromosomal sequences from autosomal sequences, but also strongly MSL-bound from weakly MSL-bound sequences. Expressed and unexpressed genes separated in the same component as strongly MSL-bound and weakly MSL-bound genes. The strongly MSL-bound, expressed genes are expressed at slightly higher levels, on average, than the weakly MSL-bound, expressed genes (8.37 versus 7.44, respectively). However, weakly MSL-bound but expressed genes cluster with unexpressed genes. Although we grouped genes into single observations our results show that all gene features have sequence signatures that could potentially be involved in MSL-complex targeting. When we studied the sequence words enriched in MSL complex-bound genes identified by each of the gene feature PCA models we found that intron, 3' UTR and 5' UTR sequences were GA, CA or adenine enriched whereas intergenic sequences were guanine and cytosine enriched (Additional file 2).

### Promoters and coding sequences are potentially involved in MSL-complex spreading along X chromosomes

We next wanted to see whether it was possible to identify sequences or motifs that could be used to predict the MSL-binding status of individual genes. For this, we applied the supervised multivariate method Orthogonal Partial Least Squares Discriminant Analysis (OPLS-DA) [38,39], which seeks variables that are predictive of a pre-defined classification of the observations (rather than merely overrepresented). Since transcription has been shown to be important for MSL-complex targeting [17], we excluded all genes for which transcription levels cannot be accurately determined (see Methods). We selected genes that are expressed and strongly bound by the MSL-complex (n = 167) and those expressed but weakly bound by the MSL-complex (n = 151) as before. In the different models some genes were excluded because they lacked the annotated gene feature modeled (see Methods and Additional file 7). Two-thirds of the dataset was randomly selected and used as a training set for constructing the models, and the other third as a test set for assessing the accuracy of their predictions. We excluded intergenic sequences as they cannot be specifically assigned to a particular gene. We obtained significant models for promoters, CDS and introns, but not for 3' UTRs and 5' UTRs (Additional file 3). By plotting the relation between the first component and both expression levels and AT-content we determined that expression levels did not significantly influence the models (Additional file 8). However, AT-content strongly affected the intron and 3' UTR models and when we normalized for AT-content, we obtained no significant models for these features. The promoter and coding sequence models were then used to predict the MSL-binding status of the previously excluded third of the genes. Strikingly, the Y prediction scores for expressed genes strongly and weakly bound by MSL differed significantly according to both the promoter and coding sequence models (Mann-Whitney *U *Test; *p *= 3 × 10^-6 ^and 1 × 10^-3^, respectively). To verify these results we constructed new models, for which we again randomly selected two thirds of the data for modeling and one third for testing predictions. The prediction results were very similar (data not shown). The promoter and coding sequence models could therefore robustly predict the MSL-binding status of the genes.

### Insulator protein BEAF-32 is enriched in promoters of genes strongly bound by MSL

**Figure 3:**
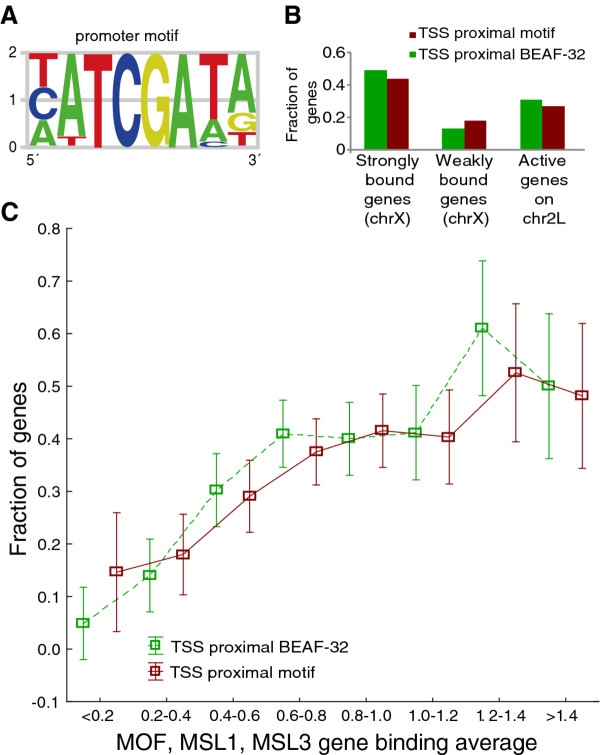
**Results from promoters of MSL-bound genes**. Results based on OPLS-DA of promoter sequences of genes expressed and strongly or weakly bound by MSL complex on chromosome X. **(A) **Promoter motif built from top sequences words. **(B) **Fraction of genes strongly and weakly bound by MSL as well as expressed genes on chromosome 2L with the promoter motif and BEAF-32 binding within 500 bp of the TSS. **(C) **Fraction of all expressed genes on the X-chromosome with the promoter motif (red) or BEAF-32 binding (green) within 500 bp of TSS grouped by average MSL enrichment (average of MSL-1, MSL-3 and MOF gene binding values). Note that a weakly bound gene in the initial model was a gene that had gene binding values for all the three proteins lower than 0.5. 90% of these genes fall into the classes with average MSL enrichment less than 0.4. The average MSL enrichment for the weakly bound class is 0.24

Encouraged by the promoter sequence modeling results, we wanted to know whether the presence of MSL is associated with any specific DNA motifs. We therefore developed an iterative algorithm that aligns the predictive sequence words from a model into motifs. The most predictive sequence words are given stronger weighting during the motif construction, and the algorithm assures that the predictive power of the evolving motif is maintained or increased (see Methods). Using this algorithm we extracted the motif shown in Figure 3A from the model based on two thirds of the promoter dataset (Additional file 7). This motif scored significantly higher in promoters (sequences extending 500 bp upstream of TSS) of strongly MSL-bound, expressed genes than in promoters of weakly bound, expressed genes when using the previously excluded test set (Mann-Whitney *U *Test *p *= 7.3 × 10^-3^). We then mapped the motif across the entire X chromosome and calculated the average distances to transcription start sites of expressed genes strongly bound and weakly bound by the MSL-complex. We found the motif to be closer to the TSS of strongly MSL-bound genes on average than to those of the weakly bound, expressed genes (Mann-Whitney *U *Test *p *= 6.9 × 10^-6^). The motif was found within 500 bp of the TSS in 44% of the strongly and 18% of the weakly bound genes. The corresponding number for active genes on chromosome 2L is 27% (Figure 3B). We also tried to construct motifs using predictive sequence words for weakly MSL-bound genes. Since this was unsuccessful we conclude that there are no clear motifs in promoters that could potentially block MSL-recruitment.

Our OPLS-DA model was based on two-thirds of the 167 strongest bound genes on the X-chromosome and previous studies have indicated that 534-773 genes are bound by the complex [17,19,20]. Therefore, we plotted the fraction of genes containing the promoter motif within 500 bp of the TSS versus the average gene-binding value of the three MSL proteins for all expressed X-linked genes (Figure 3C, Additional file 9). We observe that promoters of genes that have an average binding of the three MSL-proteins considered here of about 0.6 or more seems to have an enrichment of this motif. The number of expressed genes with an average of the three proteins above 0.6 is 660, which falls within the range of previous estimates for number of MSL bound genes. Further, a search with our promoter motif PWM in the TOMTOM tool [40] indicated that the promoter motif match the boundary element associated factor BEAF-32 motif. To test whether this protein preferentially binds to promoters of MSL-bound, expressed genes we downloaded BEAF-32 mapping data gathered by the modENCODE consortium for S2 cells [41]. Interestingly, the average distance to a BEAF-32 binding site from the transcription start site is significantly shorter for strongly MSL-bound, expressed genes than for both weakly MSL-bound, expressed genes (Mann-Whitney *U *Test *p *= 2.6 × 10^-18^) and unexpressed genes (Mann-Whitney *U *Test *p *< 1 × 10^-20^). In addition, a larger fraction of the strongly bound class of genes have BEAF-32 binding within 500 bp of the TSS (49%) compared to weakly bound genes (13%) and active genes on chromosome 2L (31%, Figure 3B). Similar to the motif, the BEAF-32 binding sites overlap a larger fraction of the genes (within 500 bp of the TSS) that have an average gene-binding value of the three MSL proteins of more than about 0.6, likely representing functionally MSL-bound and dosage compensated genes (Figure 3C, Additional file 9). However, we found no general enrichment of BEAF-32 on the X chromosome relative to the autosomes when we analyzed genome coverage of binding regions (3L has the lowest, 2R the highest and X chromosomes intermediate coverage: 1.6%, 2.0% and 1.9%, respectively). To test whether only BEAF-32 is found preferentially upstream of strongly bound genes or whether also other insulator proteins behave similarly, we run the same analysis (distance to TSS) using CP190, Su(Hw) and CTCF (also mapped by modENCODE consortium for S2 cells [41]). None of the other insulator proteins showed any preference for genes strongly bound by MSL (Additional file 10). However, Su(Hw) has a tendency to bind upstream of expressed but weakly MSL-bound genes (Mann-Whitney *U *Test *p *= 7.5 × 10^-4^).

**Figure 4:**
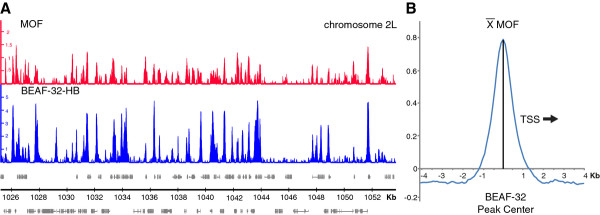
**Comparison of MOF and BEAF-32 binding sites**. **(A) **MOF (red) and BEAF-32 (blue) log_2 _enrichment profiles in a typical region of chr2L. Scale bar show the genomic positions in Kb. Genes expressed from left to right are shown above the horizontal line and the genes expressed in the opposite direction are shown below the line. **(B) **The average MOF enrichment values in 100 bp bins surrounding promoter peaks of BEAF-32 in autosomes. Promoters where aligned so that the TSS will always be to the right of the BEAF-32 peak in the figure.

It is known that MOF, but not the other components of the MSL-complex, targets the promoters of MSL-complex bound genes [20]. We therefore wanted to test whether the binding of MOF to promoters correlates to the binding of BEAF-32. Both MOF and BEAF-32 binds to promoters of active genes also on autosomes [20,42]. Since MOF binding levels are very different between the X-chromosome and the autosomes, we focused on the larger autosomal data set. Visual inspection of the MOF and BEAF-32 profiles clearly indicate that both proteins co-localize in promoters (Figure 4A). When we study autosomal distribution of BEAF-32, 99.5% of all binding regions have MOF enrichment above background levels (average autosomal enrichment). 97% of all BEAF-32 binding regions have MOF enrichment more than background plus one standard deviation. We next selected the BEAF-32 regions overlapping promoters on autosomes and plotted the average MOF enrichment values in 100 bp bins surrounding the BEAF-32 peak center (sequences where oriented so that the direction of transcription was the same). It is clear that the MOF binding peaks at the same position as the BEAF-32 peak center (Figure 4B). Although, the MOF enrichment is low at some BEAF-32 sites we conclude that MOF and BEAF-32 strongly correlate across the *Dm *genome.

The High Affinity Sites (HAS) that are thought to be responsible for the initial recruitment of the MSL-complex to the X-chromosome, was defined by being the strongest MSL-sites along the X chromosome [29]. Therefore, it is likely that many of the genes we selected as strongly bound (the genes along X with the strongest gene-binding values) overlap a HAS. To find out how our results correlate with the presence of high affinity sites we used 188 defined HAS (a union of the HAS reported by Alekseyenko et al. [18] and Straub et al. [22]. Indeed, 116 of the 167 strongly bound genes overlap or have a HAS within 5 kb. We also see that the top sequence words from the very weak 3' UTR OPLS-DA model with two-third dataset (Additional file 7) resembles the GA-rich HAS motif [18,22]. To investigate if the presence of the promoter motif we identified is correlated to the presence of HAS, we divided the strongly bound genes into those proximal to a HAS (within 5 kb) and those far from a HAS (> 5 kb). We found no significant difference in terms of distance from the TSS to the nearest promoter motif for these two groups (Mann-Whitney *U *Test *p *= 0.90). In fact, 41% of the HAS proximal strongly bound genes have the motif within 500 bp of the TSS compared to 49% of the strongly bound genes far from a HAS (> 5 kb). We also constructed a new promoter OPLS-DA model (genes strongly bound by MSL versus genes weakly bound by MSL), this time excluding genes within 5 kb of a HAS. Since, many of the most strongly bound genes are close to a HAS, we decreased the cut-off for being strongly bound to 0.8 (instead of a binding value of at least 1 for the three proteins). We ended up with 86 strongly bound and 88 weakly bound genes that are far from any HAS. The motif extracted from this model is virtually identical with the motif in Figure 3A, showing that this motif is not correlated to High Affinity Sites. In addition, the expressed genes on the X chromosome with BEAF-32 in the promoter are not preferentially found close to high affinity sites (Additional file 9).

### Sequence signatures in coding sequence of genes strongly bound by MSL have a 3' bias

**Figure 5:**
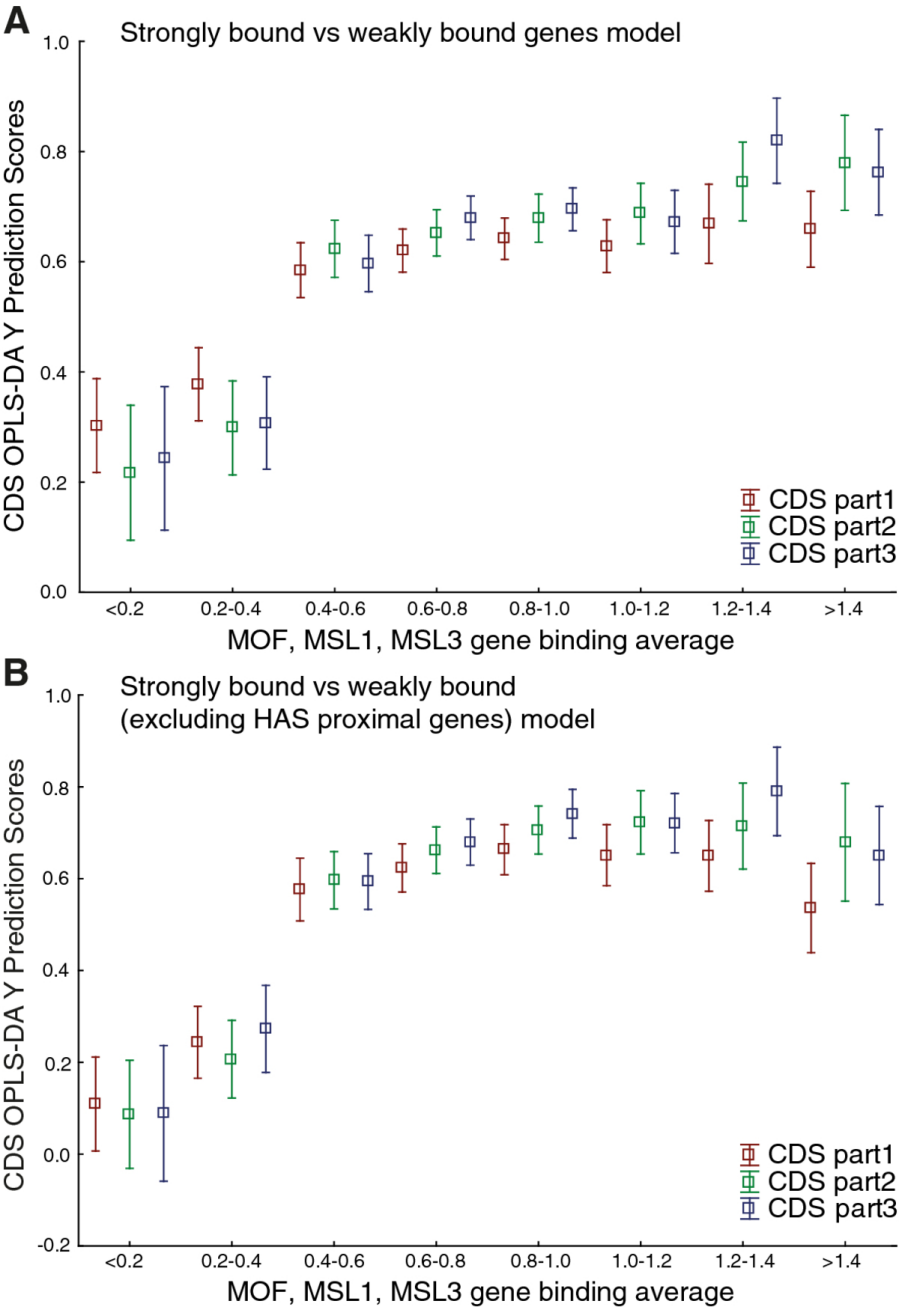
**Results from CDS of MSL-bound genes**. Results based on OPLS-DA of CDS of genes that are expressed and strongly or weakly bound by MSL complex on chromosome X. **(A) **OPLS-DA Y Prediction scores of three equally sized parts of CDS from all expressed genes on chromosome X compared to average MSL enrichment (average of MSL-1, MSL-3 and MOF binding values). OPLS-DA model based on CDS of MSL complex strongly and weakly bound genes. Note that the weakly bound genes in the model have an average binding value for the three proteins of 0.24 and 90% fall into the first two binding classes (< 0.2 and 0.2-0.4). Weakly bound genes were selected as genes that had gene binding values for all the three proteins lower than 0.5. **(B) **OPLS-DA Y Prediction scores of three equally sized parts of CDS from all expressed genes compared to average MSL enrichment. OPLS-DA model based on CDS of MSL complex strongly and weakly bound genes 5 Kb away from any HAS.

Using our modeling approach we found sequence variations in coding sequence that are strongly predictive of MSL-bound, expressed genes. However, our sequence word aligner failed to extract any complex motifs from the coding sequence model (for either strongly bound or weakly bound genes). We concluded that, as expected, there are no long, complex MSL-targeting motifs in coding sequences, but when we calculated the frequencies of sequence words in the analyses described above we merged the scores for the forward and reverse complements of each word. Thus, we scored both strands of each sequence region. Therefore, for completion we examined the possibility that predictive sequence words for genes strongly bound by MSL may be preferentially located on only one strand and/or preferentially in-frame. Models based on sequence word frequencies in the transcribed strand only, or only in-frame, did not perform as well as the original model for predicting the excluded third of the dataset (Additional file 3). We conclude that the short sequence words that are predictive of strongly MSL-bound, expressed genes are not preferentially located either on transcribed strands or in-frame. Further, as the X chromosome is known to have a more optimal codon usage than the autosomes [43], we tested whether there are any significant differences in codon usage between expressed genes that are strongly bound and weakly bound by the MSL-complex. We found evidence of codon usage bias between Chromosomes X and 2L (Paired *T*-Test *p *= 9.5 × 10^-5^), but not between expressed MSL complex strongly bound and weakly bound genes (Paired *T*-Test *p *= 0.27). However, the MSL-complex is known to preferentially bind to exons with a bias towards the 3' end of the genes, and excitingly, when we divided the coding sequences of the genes in the prediction set into three equally sized parts, the MSL binding status was significantly better predicted for their 3' ends than for their 5' ends (Paired *T*-Test *p *= 2.5 × 10^-3^), and the binding status of the middle parts of the coding sequences were better and less well predicted, on average, than that of their 5' and 3' parts, respectively. We also constructed an OPLS-DA model using the complete coding sequence of all strongly bound (n = 167) and weakly bound (n = 151) genes and then used this model to predict the coding sequence of all expressed genes on X (n = 973), divided into three equally sized parts. Again, the 3' part of the coding sequence of genes with an average binding level of the three MSL-proteins of about 0.6 is better predicted when compared to the 5' part (Figure 5A). As for the promoter model we wanted to know whether the coding sequence model was strongly influenced by High Affinity Sites. We selected strongly bound and weakly bound genes that did not have a HAS within 5 kb of either end. Performing OPLS-DA using these genes resulted in a similar model as before and in Figure 5B is shown the prediction of the three parts of coding sequence of all expressed genes on the X-chromosome (to be compared to Figure 5A, where genes close to a HAS was also included in the model). Hence, the distribution of the MSL-complex within genes is most likely influenced by sequence signatures in the coding sequence. For a summary of the promoter and CDS results for expressed genes along the X chromosome see Additional file 9.

Since the number of genes in the initial model is relatively low (around 200-300 observations) we wanted to test how likely it is that a model based on similar numbers of randomly selected genes produces a significant OPLS-DA model. We randomly selected seven non-overlapping groups consisting of coding sequence from 100 expressed genes from chromosome 2L. Using OPLS-DA we modelled all pair wise combinations of these seven groups (21 combinations). All pair wise combinations produced models with negative Q2 values (predictive power). Six models had a strong correlation to AT-content (Spearman R > 0.4). Normalizing for AT-content did not improve the models. Next, we repeated this process with coding sequence from seven randomly selected groups of intermediately bound genes (100 genes/group) on the X-chromosome. Intermediately bound genes where defined as expressed genes on the X chromosome not belonging to the previously defined strongly and weakly MSL-bound genes. 20 pair wise combinations produced models with a negative Q2 and one combination produced a very weak model with a Q2 of 0.03 (to be compared to a Q2 of 0.27 in the strongly versus weakly MSL-bound model). Three models had a strong AT-correlation, but normalizing for nucleotide content did not improve the models. We conclude that randomly selected groups of sequences are unlikely to produce a significant model with predictive power.

## Discussion

We have thoroughly investigated X chromosome sequence variation in *D. melanogaster *and related this variation to the targeting of the dosage compensation complex, using frequencies of two to six base pair sequence "words" and multivariate statistical analyses. The advantage of our approach is that it is unbiased and focused on finding sequences with predictive value, rather than merely over-represented sequences. First, we divided the genome sequence into intergenic, promoter, 5' UTR, coding, intron and 3' UTR sequences. Interestingly, there is more divergence among these six sequence types or gene features than within the sequence types on different chromosomes (see Figure 1). Our findings also show that sequences are present in promoters and coding sequence that could be involved in the spreading of the MSL-complex from the high affinity sites on the X chromosome. The coding sequences we have identified share a similar 3' bias with the MSL-complex. Further, the highest scoring promoter sequences form the target motif of the insulator protein BEAF-32, and BEAF-32 mapping data indicate that this protein binds preferentially upstream of genes strongly bound by MSL.

### Sequence variation between gene features

Different gene features are known to vary in sequence composition, but in our opinion their variation is not normally taken into account in attempts to discover new sequence motifs. We here show the extent of this sequence variation, and that coding sequences have the most distinct sequence composition followed by 5' UTRs, 3' UTRs and promoters. This has important implications for studies of sequence variation and motif discovery; when groups of sequences are compared it is important to take gene features into account (*e.g*. when using the MEME option of discriminative motif discovery), otherwise the results may reflect differences in gene feature composition rather than biologically relevant sequence variation.

### The X chromosome has a distinct sequence composition

Our separate analyses of the six gene features clearly show that the sequence composition of those in the X chromosome differs from the composition of corresponding features in all other chromosomes (Additional file 4). This distinction of the X chromosome is mainly due to differences in frequencies of various di-nucleotides, many of which have been previously found to be enriched on X [33,34]. These sequences could, in principle, be involved in recruiting X chromosome-specific factors, such as the MSL-complex. Apart from being dosage-compensated in males, the X-chromosome might also be under selective forces that do not act on the autosomes. Some of the sequence variation of the X-chromosome is likely a result of its evolution as a sex chromosome. The MSL-complex is the only known protein complex involved in dosage compensation in *Drosophila* with an X chromosome-specific distribution. We have focused here on the sequence variation that could be related to the targeting of this complex. It has been shown that the MSL-complex is initially targeted to X by binding to so-called high affinity sites (HAS) that contain the GA-rich MSL recognition element (MRE) [18,22]. The MSL-complex can be recruited to autosomes by inserting MRE-containing high affinity sites [18,22], but the mechanism involved in the spreading of MSL to X-chromosomal genes is under debate [15,44]. We have investigated here whether sequence patterns may be involved in this spreading of the MSL-complex, as discussed below.

### Genes strongly bound by MSL have a distinct sequence composition

The genome distribution of the MSL-complex has been mapped in several studies [17-22]. We used the data from (Kind et al., [20]) to select genes that are expressed and strongly MSL-bound, expressed and weakly MSL-bound as well as unexpressed genes. This is the only currently available dataset where mapping of several MSL-complex components and transcription in mutants/knock-downs of MSL-components was done in parallel and in the same cell-type. When merging all strongly MSL-bound expressed genes into one observation and all weakly MSL-bound expressed genes into another, we find that all six sequence types have sequences that differ between strongly bound and weakly bound genes (Figure 2). We observed that sequence variation between expressed genes strongly bound and weakly bound by MSL complex is much higher than that between expressed and unexpressed genes on chromosome X. Further, expressed genes that are weakly bound by the MSL complex group more closely to unexpressed genes than to expressed MSL-bound genes in our PCA score plots. Therefore, the small but significant expression difference detected between the expressed genes that are strongly bound and weakly bound by the MSL complex did not have any major correlation on the sequence variation observed between the two groups. Sequence words extracted from PCA models of intron, 3' UTR and 5' UTR sequences were more GA, CA or adenine rich which are in agreement with the previous identification of CA dinucleotide repeats, runs of adenines and GA-rich MRE motif from High Affinity Sites (HAS) [18,22,32,35]. We conclude that there are differences in sequences of all six features between expressed genes that are strongly bound and weakly bound by the MSL-complex. However, these results merely identify sequence words that are overrepresented in groups of genes strongly or weakly bound by MSL. In order to search for predictive sequence patterns for MSL-binding to individual genes we applied Orthogonal Partial Least Squares Discriminant Analysis, OPLS-DA.

### Promoters and coding sequences are potentially involved in MSL-complex spreading along X chromosomes

Using OPLS-DA we explored differences between features of individual genes that are strongly MSL-bound and expressed versus weakly MSL-bound and expressed, extracted sequence words with the highest predictive power, and attempted to combine them into more complex motifs using the algorithm described herein. Interestingly, both coding sequence and promoter models yielded sequence words that could be used to predict the MSL-binding status of genes excluded from the modeling. Neither nucleotide content nor expression level significantly influence these promoter and coding sequence models and the top sequence words we identified are only weakly overrepresented on the X-chromosome. We conclude that promoters and coding sequences contain sequence signatures that are potentially involved in the spreading of the MSL-complex from high affinity sites. In principle, there may be motifs in unbound, expressed genes that block the binding of the MSL-complex, but we obtained no evidence for such motifs.

### Insulator protein BEAF-32 preferentially binds to promoters of MSL-bound genes

From the promoter model we extracted the motif in Figure 3A, which could be used to predict promoters of genes strongly bound by MSL. This motif proved to correspond to the targeting motif for the insulator protein BEAF-32 [45], which binds to hundreds of sites across the genome, generally located upstream of active genes [46,47]. Although the molecular mechanisms of BEAF-32 activity are unknown, it seems to be linked with active transcription [41,42]. In order to test whether the BEAF-32 protein itself is enriched at strongly MSL-bound genes we used BEAF-32 ChIP-chip mapping data obtained from modENCODE, and found that BEAF-32 preferentially binds proximal to transcription start sites of genes strongly bound by MSL. This exciting link between BEAF-32 and dosage compensation is supported by the observation that *beaf-32 *mutants have a male-specific defect in X-chromosome morphology [48]. Further, Laverty et al. [49] found that reporters inserted on the X chromosome are better able to recruit the MSL-complex if they have binding sites for GAGA and DREF factors. The DREF binding site is very similar to the BEAF-32 binding site and although DREF might be involved in dosage compensation it is possible that increased BEAF-32 recruitment is the true case of the effects observed by [49]. However, since DREF has not been mapped genome wide we cannot exclude the possibility that our promoter motif correlate better with DREF. BEAF-32 is associated with active transcription and might facilitate the MSL-complex targeting of active genes. Since MSL-complex bound genes show MOF binding in the promoter [20] and MOF clearly co-localize with BEAF-32, we hypothesize that BEAF-32 and MOF interact in promoters of MSL-complex bound genes. BEAF-32 is a DNA-binding protein and might recruit MOF to active genes on the X-chromosome, genes that are then targeted by the MSL-complex. However, further experimental efforts are needed to understand the link uncovered here between BEAF-32 and the MSL-complex.

### Sequence signatures in coding sequences of MSL-bound genes have a 3' bias

The finding of sequence patterns that are predictive of MSL-binding genes within coding sequences is intriguing, although it has been previously reported [36]. Scoring the sequence words only in the transcribed strand or the correct frame did not improve the coding sequence model, suggesting that the relationships are not attributable to (for instance) specific variations in amino acid composition. Neither did we find any codon usage bias between strongly bound and weakly bound expressed genes, or any model correlation with expression and AT-content. However, we found using OPLS-DA that bound coding sequences are rich in AG di-nucleotides, which have been previously reported to be abundant in dosage-compensated chromosomes [31].

The MSL-complex binds to genes with a preference for exons [19]. The relatively low binding to introns might suggest that the complex targets spliced RNA transcripts. However, it was recently found that the complex targets chromatin rather than transcribed RNA [50]. The exon specificity could be explained by various chromatin factors, nucleosome density and/or sequence specificity. Variations in nucleosome density may partially explain the exon bias, as it is higher in exons [51] and thus may provide more targets for H4K16 acetylation, a modification that is strongly linked to the MSL-complex [52]. In addition, the MSL-complex binding profile clearly shows that it binds most strongly towards the 3' end of genes [19]. Accordingly, our models predicted the MSL-binding status of genes better from the 3' thirds than from the 5' thirds of the coding sequences. This is in contrast to the lack of 3' bias of the [G(GC)N]_4 _motif reported by [36]. Taken together, our results strongly indicate that the MSL-complex distribution within genes on the X-chromosome is influenced by the primary DNA sequence.

## Conclusions

The MSL-complex evidently targets a limited number of High Affinity Sites along the X-chromosome. Although, many strongly bound genes are close to a HAS neither our promoter motif nor our coding sequence signatures show any correlation to HAS. Based on the results presented here, we believe that there are sequences in the promoters and coding sequences of targeted genes that have the potential to direct the secondary spreading of the complex to nearby genes. However, a number of genes are dosage-compensated by MSL-independent mechanisms [5] and expression on the X-chromosome is only reduced to ~80% of wild type levels in males when *msl *genes are mutated or knocked down using RNAi [20,24]. Apart from the dosage compensation mediated by the MSL-complex there is evidence for a more general buffering system that targets haploid regions in the genome [53]. So other, as yet unknown, factors are likely involved in compensating the X chromosome and these factors could potentially act on a number of levels, such as transcription regulation, mRNA export, mRNA stability and translation [3]. The observed optimal codon usage on the X-chromosome likely represents compensation on the translational level. However, even if additional factors involved in dosage compensation remain to be discovered, we here show that there are plenty of sequences within all types of gene features that could act as X-targeting elements.

## Methods

### Genome annotations and sequences

We obtained *Dm *genome annotation and sequence Release 5.23 from Flybase [54] and parsed non-overlapping coordinates of the six types of gene features (promoters, 5' UTRs, CDS, introns, 3' UTRs and intergenic sequences) on each of the chromosomes in *Dm *from the genome annotation data (defining promoters as sequences extending 500 bp upstream of transcription start sites). We then used the gene feature coordinates to extract corresponding sequences from the genome sequence.

### Oligonucleotide scoring

For each gene feature we constructed two-dimensional data matrices with full-length chromosomes or gene-specific regions as objects (rows) and frequencies of all possible di-(16), tri-(64), tetra-(256), penta-(1024) and hexa-mers (4096) in the gene feature sequences as variables (columns). To calculate frequencies of oligonucleotides (sequence words) we counted every word in each target sequence sliding one nucleotide at a time and divided the count by the length of the target sequence. Both forward and reverse complements of each sequence word were scored and treated as one.

### Determining expression and MSL-binding of genes

To explore the relationship between sequence variation in X chromosome gene features and targeting of the MSL complex to X chromosomes we used the SL-2 cell ChIP-chip and gene expression array data presented by [20]. We preprocessed raw Affymetrix (ChIP-chip MSL-1, MSL-3 and MOF) .CEL files using Tiling Analysis Software (TAS; http://www.affymetrix.com/partners_programs/programs/developer/TilingArrayTools/index.affx), with default parameters and Probe Analysis settings: bandwidth (BW) - 300, Test Type - one sided upper, perfect match probe intensities. We used the Integrated Genome Browser (IGB; [55] to visualize data, then converted the genome coordinates of Release 4 to those of the latest Release (5), using the Flybase sequence coordinate converter tool. We considered all probes in all exons within each gene and calculated gene binding values as the average of the top 50% probes, to minimize the influence of alternative splicing. Genes on the X chromosome with enrichment ratios exceeding one (in log_2 _scale) for all three proteins were selected as representatives of genes strongly bound by the MSL complex and genes with enrichment ratios of the three proteins below 0.5 as representatives of weakly bound genes. These cut-offs was set in order to select the two extremes in terms of binding values, while keeping the size of the two groups similar as well as reasonably large. Genes with enrichment ratios between 0.5 and one were excluded from the initial models (Additional file 11 and Additional file 12). For the different gene feature models we excluded genes that lacked the gene feature in question and for the promoter models we excluded genes for which their entire 500 bp upstream region overlapped a neighbouring gene (promoters with partial overlap to other genes or promoters were truncated).

Gene expression levels in EGFP control SL-2 cells relative to *mof, msl-1 *and *msl-3 *RNAi knock-down cells were computed from raw gene expression Affymetrix .CEL files using Robust Multi-Array Average (RMA) [56] with the Bioconductor "affy" package [57]. Using the latest library files from Affymetrix we then mapped each probeset to genomic release 5 coordinates. For each gene, expression was calculated as the median of the three replicates. Any effect on gene expression after RNAi knock-down can be observed only for genes expressed between six and 10 in EGFP control after RMA normalization (Additional file 13). This means that genes with expression levels outside this range are either unaffected by the RNAi or their expression levels cannot be accurately estimated. Hence, we focused on genes with expression values between six and 10 and considered those with expression values less than six as unexpressed.

### Determining insulator proteins binding sites

Insulator proteins (BEAF-32, CP190, CTCF and Su(Hw)) S2 cell-line ChIP-chip data from modENCODE (BEAF-HB.S2, BEAF-70.S2, CP190-VC.S2, CP190-HB.S2, CTCF-VC.S2, CTCF.S2 and SU(HW)-HB.S2, Su(Hw)-VC.S2) were preprocessed as described above. To identify bound regions, and their peak positions, binding ratios exceeding the genomic average by at least three standard deviations were extracted. Bound regions were then defined by stretches of array probes (passing the three standard deviation cut-off) at least 360 bp in length and a region was extended as long as there was a value within 360 bp of the previous value. Regions with fewer than five probes were excluded. The value for each detected region was set to the average of the highest six consecutive probe values (ratios). The center of each peak was set to the mid-position of the six highest consecutive probe values. Only binding regions detected in both datasets (obtained using two different antibodies for each insulator protein) were used. For each protein, distances to transcription start sites were calculated as the distances from each TSS to the nearest peak center of each protein.

### Statistical analysis

All multivariate analysis and visualization described below was performed using Evince (Umbio, Sweden), except OPLS analysis, for which we used SIMCA (Umetrics, Sweden). AT normalization was applied by dividing each word frequency by the expected frequency based on the nucleotide composition of the word and the target sequence assuming random distribution of nucleotides [34]. We center-scaled all data that were AT normalized prior to multivariate analysis. For all other modeling we applied unit variance (UV) scaling.

All univariate statistical analysis and visualization were performed using Statistica software (Statsoft, USA).

### Sequence word alignment and motif scoring

We developed an iterative algorithm to identify complex motifs with significant predictive values for MSL complex binding, based on the top sequence words obtained from OPLS-DA models designed to detect sequence differences between genes strongly bound and weakly bound by the complex. A flowchart for the algorithm is presented in Additional file 14, and *Perl *codes for the sequence word aligner can be downloaded from http://www.molbiol.umu.se/english/research/researchers/per-stenberg/. This algorithm constructs motifs in the form of position weight matrices (PWMs), constructed by summing loadings of sequence words included in the motif and normalizing so that values in each column sum to one. Each motif is scored at all possible positions in each sequence by multiplying the values of each matching position in the PWM. A user-defined number of best scores (here one) within each sequence are then summed. The top 1000 sequence words (sorted by loading) from each OPLS-DA model were used in the alignment presented here. If a sequence word is used to extend the PWM it is excluded from further extensions. All sequence words are tested for variation of the PWM before each extension. Both forward and reverse complements of the sequence words are tested in each alignment and up to two simultaneous alignments to the PWM are tested. Logistic regression is used to test each update of the PWM for predictive improvement. When the predictive value of each PWM can no longer be improved, the resulting motif is masked in all sequences and a new PWM is created from the top sequence word in the remaining list of sequence words (excluding sequence words included in any previous PWM).

We defined a cut-off score for the promoter motif using the highest scores of the motif in each of the promoters of expressed genes strongly and weakly bound by MSL, such that the motif would be present in only 5% of the expressed, weakly bound genes. This cutoff was used to find promoter motif sites across the X chromosome.

All custom software and scripts are available upon request.

## Abbreviations

*Dm*: *Drosophila melanogaster*; MSL: Male-specific lethal; CDS: Coding Sequence; 5' UTR: 5 prime untranslated region; 3' UTR: 3 prime untranslated region; TSS: Transcription start site; HAS: High affinity site; PCA: Principal component analysis; OPLS-DA: Orthogonal partial least squares discriminant analysis; PWM: Position weight matrix

## Competing interests

The authors declare that they have no competing interests.

## Authors' contributions

All authors participated in the design of the study. PP and FP carried out the implementation and the analysis. PP and PS wrote the manuscript. All authors read and approved the final manuscript.

## Supplementary Material

Additional file 1 **Gene feature AT-contents**. AT contents of indicated gene features in Dm chromosomes. Table cells colored according to chromosome AT content (low - red to high - green). Click here for file

Additional file 2 **Sequence words from PCA models**. Sorted lists of sequence words with corresponding loadings from all PCA multivariate models. Click here for file

Additional file 3 **Summary of fits for all multivariate models**. Summary of fits for all multivariate models. Click here for file

Additional file 4 **PCA of individual gene features**. Results of PCA of frequencies of sequence words within individual gene features in *Dm *chromosome arms. Chromosomes colour-coded, and gene features indicated by symbols as follows: green = X, magenta = 3R, brown = 2R, blue = 3L, yellow = 2L; ∇ = promoter, Δ = 5'UTR, □ = CDS, O = intron, + = 3'UTR, × = intergenic. (A), (B), (C), (D), (E) and (F) show 1^st ^vs 2^nd ^component score plots (R2cum = 0.473, 0.478, 0.759, 0.821, 0.565 and 0.791, respectively) of the AT-normalized 2-6 mer promoter, 5' UTR, coding sequence, intron, 3' UTR and intergenic sequence analyses, respectively. Click here for file

Additional file 5 **Correlation of gene-binding values from different data sets**. Spearman correlations of gene-binding values for MOF, MSL-1 and MSL-3 proteins from different data sets. All correlations are significant (*p *< 0.05). Click here for file

Additional file 6 **Gene feature AT-contents grouped by MSL binding and expression status**. AT contents of indicated gene features in expressed genes strongly and weakly bound by the MSL complex, and all expressed and unexpressed genes. Table cells colored according to the gene features' AT contents (low - red to high - green). Click here for file

Additional file 7 **Sequence words from OPLS-DA models**. Sorted lists of sequence words with corresponding loadings from all OPLS-DA multivariate models. Click here for file

Additional file 8 **Correlation between OPLS-DA models and AT-content as well as expression**. Results from OPLS-DA models of frequencies of sequence words in features of expressed MSL strongly bound (green, O) vs MSL weakly bound genes (magenta, O) of chromosome X. (A), (B), (C) and (D): scatter plots of first component scores versus AT contents of promoters, coding sequences, introns and 3' UTRs, respectively. (E), (F), G) and (H): scatter plots of promoter, coding sequence, intron and 3' UTR first component scores versus gene expression levels, respectively. Click here for file

Additional file 9 **Heatmap summarizing our promoter and CDS results on chromosome X**. A heatmap of expressed genes on chromosome X sorted with respect to average MSL enrichment. In the heatmap, presence of BEAF-32 within 500 bp of transcription start site (TSS) (column 2), promoter motif presence within 500 bp of TSS (column 3), OPLS-DA Y Prediction scores of three equally sized parts of CDS (columns 4-6) and high affinity site distance to genes (column 7) are shown. Click here for file

Additional file 10 **Insulator protein-TSS distances**. Average distances from transcription start sites (TSS) of expressed MSL-bound (magenta) and - unbound (green) genes to BEAF-32, CP190, CTCF and SU(HW) binding sites. Click here for file

Additional file 11 **Plot of gene-binding values of MOF, MSL-1 and MSL-3 proteins**. Enrichment of MOF, MSL-1 and MSL-3 within autosomal and X-linked genes in SL-2 cells (to the left and right, respectively) is shown by blue, red and green lines, respectively. Click here for file

Additional file 12 **Gene-binding values of MOF, MSL-1 and MSL-3 proteins**. Gene-binding values for all genes in the genome. Click here for file

Additional file 13 **Expression of X-linked genes in control and in RNAi knockdown of *msl*-genes**. Expression of X-linked genes in EGFP control (blue) and in RNAi knockdown of *mof, msl-1 *and *msl-3 *genes (red) in SL-2 cells, sorted by expression in EGFP controls. (A), (B) and (C): line plots of expression in EGFP control and *mof *RNAi, *msl-1 *RNAi and *msl-3 *RNAi cells, respectively. Expression levels in RNAi treated cells are shown as running averages for sets of 21 genes. Click here for file

Additional file 14 **Sequence word aligner algorithm**. A flowchart for the iterative algorithm to identify complex motifs with significant predictive values for protein binding, based on the top sequence words obtained from OPLS-DA models designed to detect sequence differences between genes bound and not bound by the protein. Click here for file
