# Supplementary figures and images for: Sequence signatures involved in targeting the male-specific lethal complex to X-chromosomal genes in Drosophila melanogaster
Source: BMC Genomics. 2012 Mar 19;13:97. doi: 10.1186/1471-2164-13-97 (PMC3355045; doi:10.1186/1471-2164-13-97)

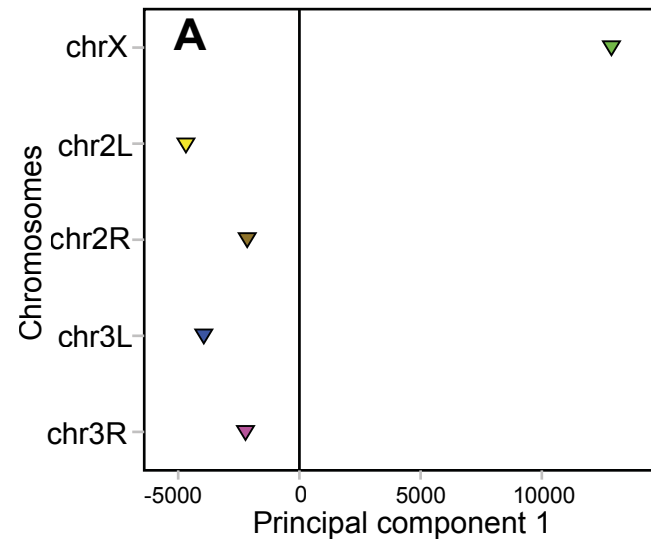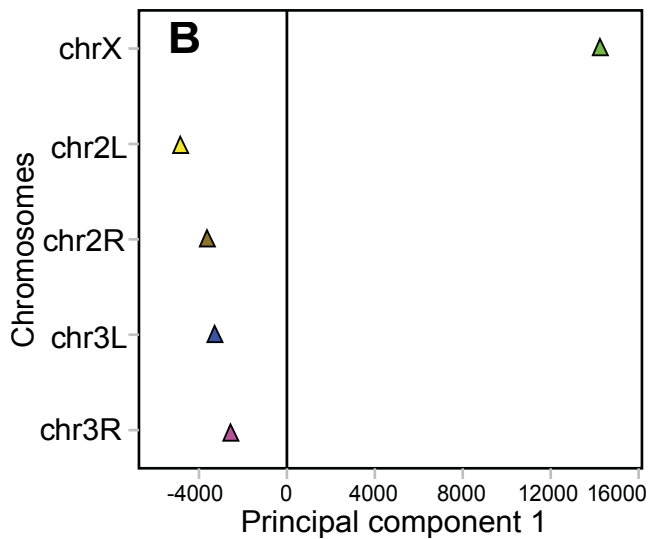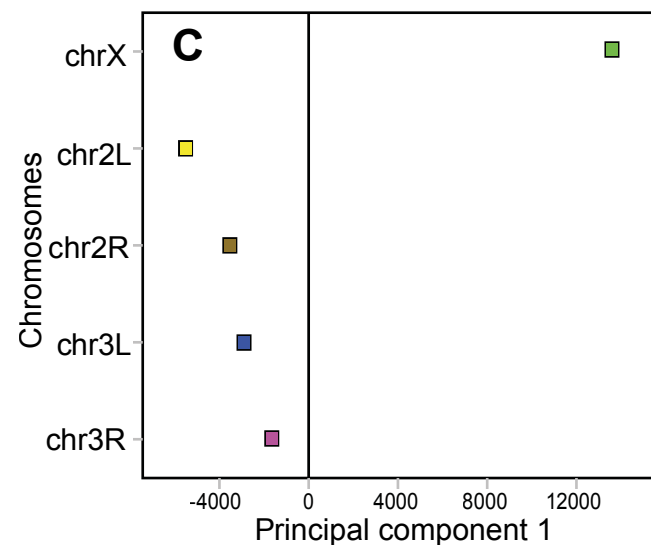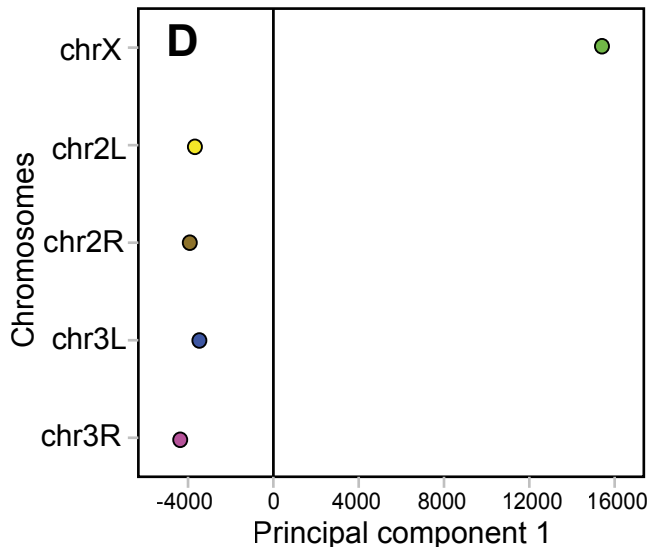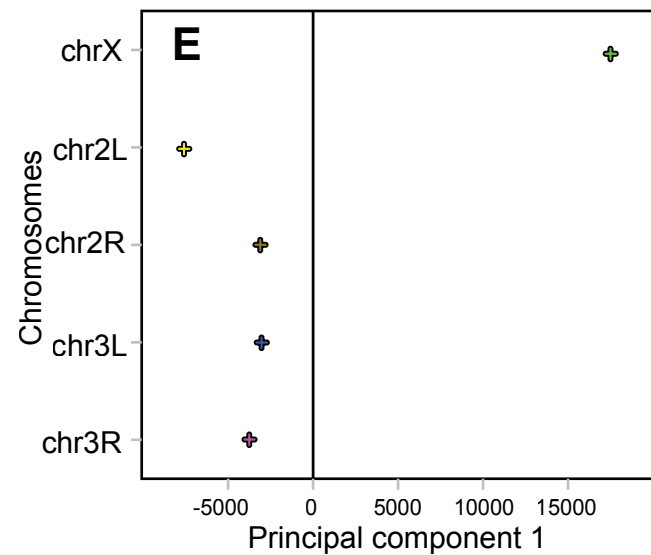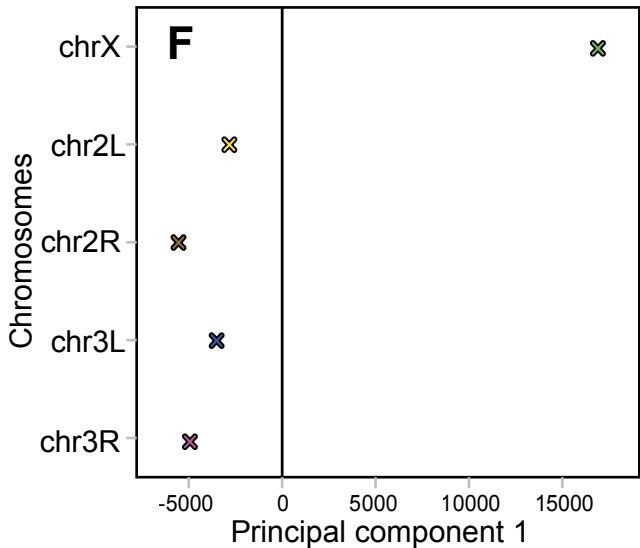

Supplement: Additional file 4 — PCA of individual gene features. Results of PCA of frequencies of sequence words within individual gene features in Dm chromosome arms. Chromosomes colour-coded, and gene features indicated by symbols as follows: green = X, magenta = 3R, brown = 2R, blue = 3L, yellow = 2L; ∇ = promoter, Δ = 5'UTR, □ = CDS, O = intron, + = 3'UTR, × = intergenic. (A), (B), (C), (D), (E) and (F) show 1st vs 2nd component score plots (R2cum = 0.473, 0.478, 0.759, 0.821, 0.565 and 0.791, respectively) of the AT-normalized 2-6 mer promoter, 5' UTR, coding sequence, intron, 3' UTR and intergenic sequence analyses, respectively. [file 1471-2164-13-97-S4.PDF]

# MOF, MSL1, MSL3 gene binding average ranges

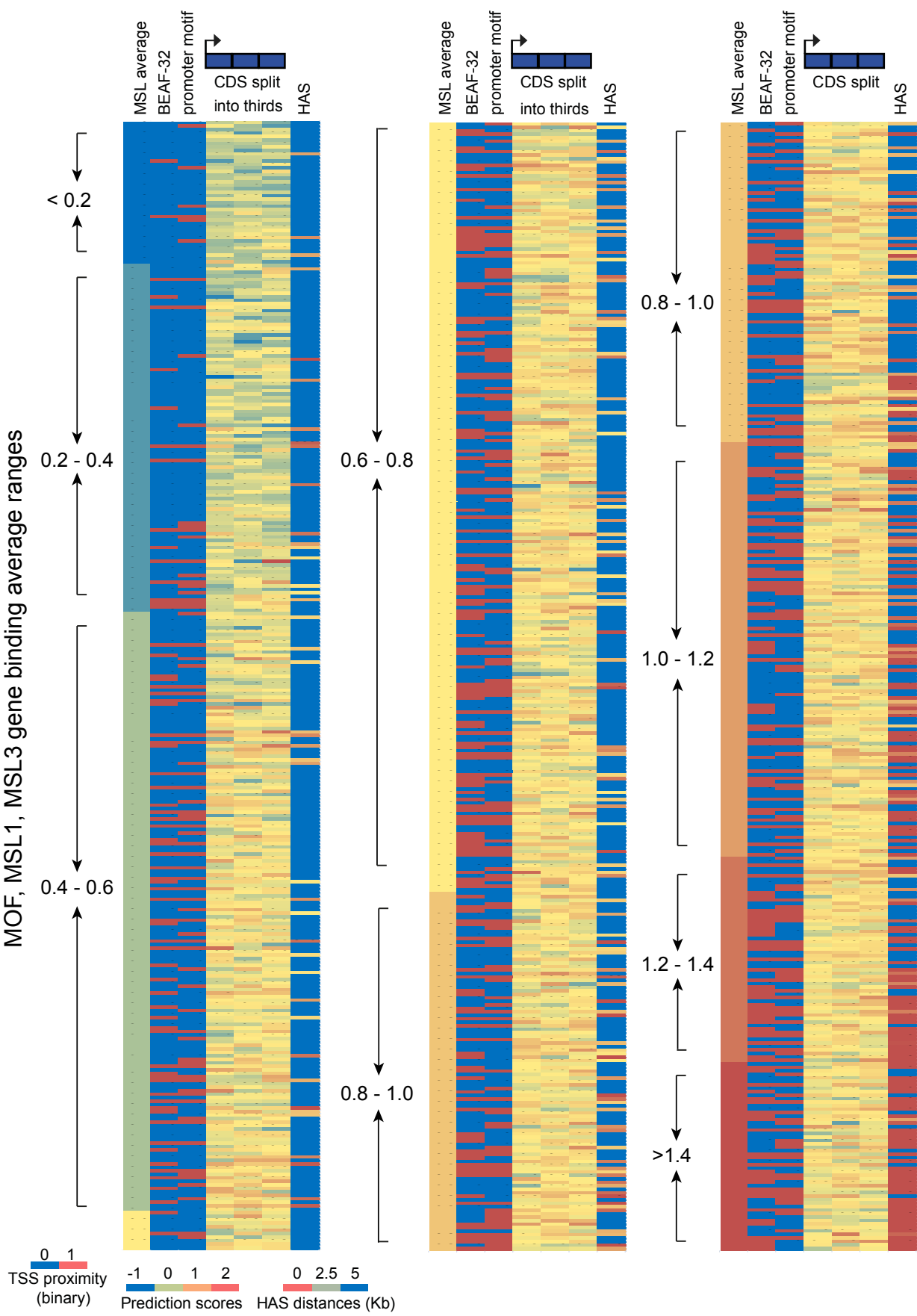

Supplement: Additional file 9 — Heatmap summarizing our promoter and CDS results on chromosome X. A heatmap of expressed genes on chromosome X sorted with respect to average MSL enrichment. In the heatmap, presence of BEAF-32 within 500 bp of transcription start site (TSS) (column 2), promoter motif presence within 500 bp of TSS (column 3), OPLS-DA Y Prediction scores of three equally sized parts of CDS (columns 4-6) and high affinity site distance to genes (column 7) are shown. [file 1471-2164-13-97-S9.PDF]

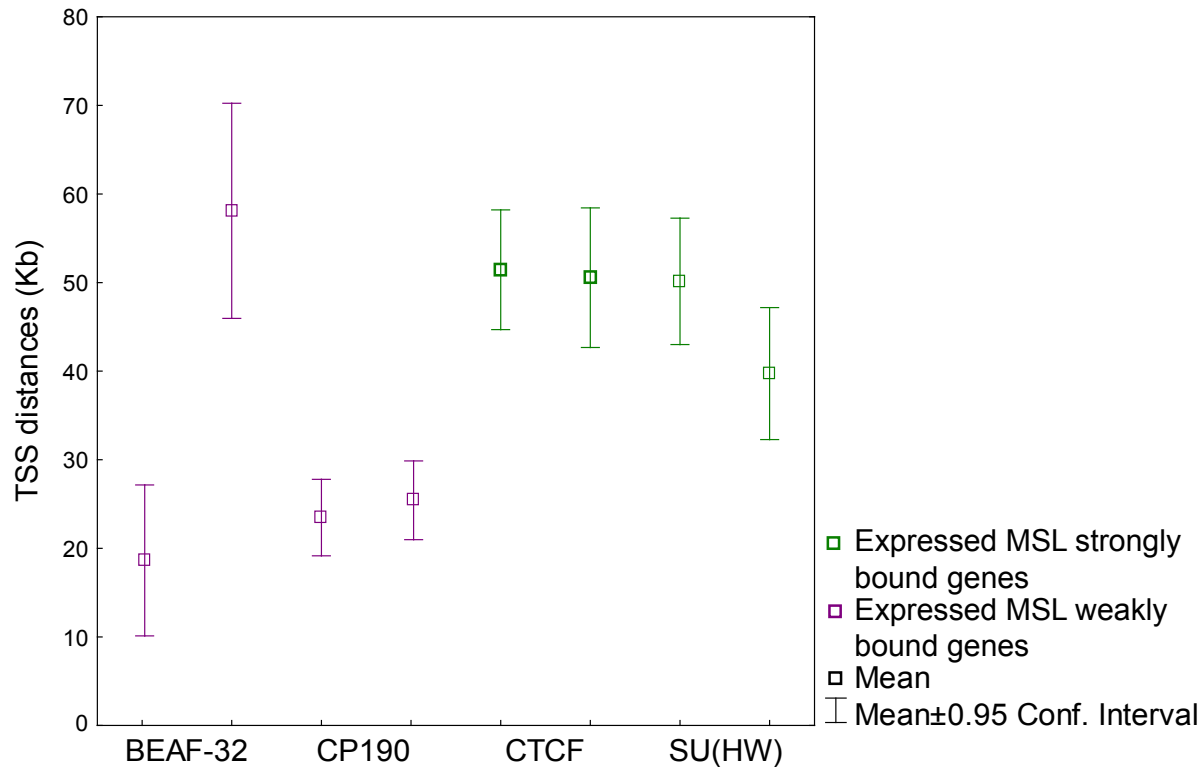

Supplement: Additional file 10 — Insulator protein-TSS distances. Average distances from transcription start sites (TSS) of expressed MSL-bound (magenta) and - unbound (green) genes to BEAF-32, CP190, CTCF and SU(HW) binding sites. [file 1471-2164-13-97-S10.PDF]

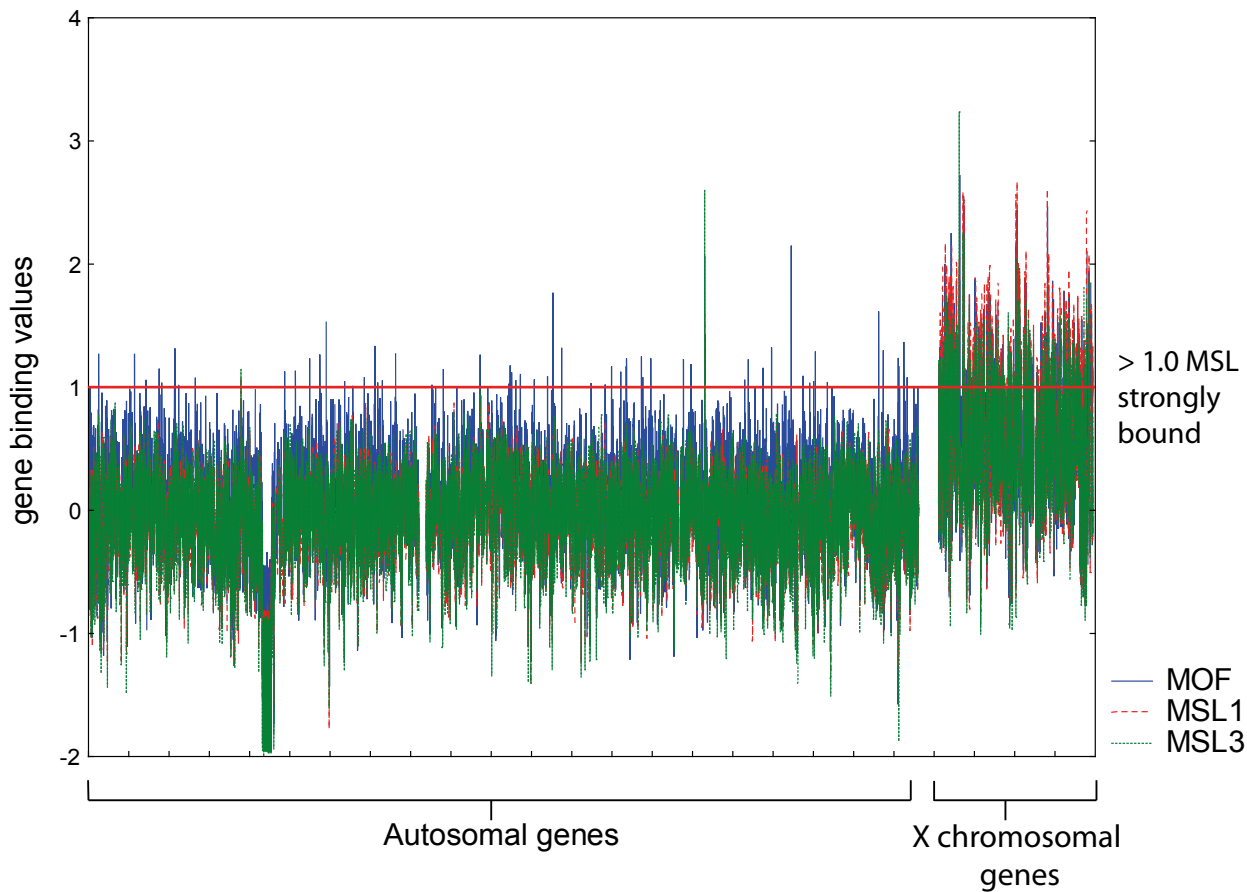

Supplement: Additional file 11 — Plot of gene-binding values of MOF, MSL-1 and MSL-3 proteins. Enrichment of MOF, MSL-1 and MSL-3 within autosomal and X-linked genes in SL-2 cells (to the left and right, respectively) is shown by blue, red and green lines, respectively. [file 1471-2164-13-97-S11.PDF]

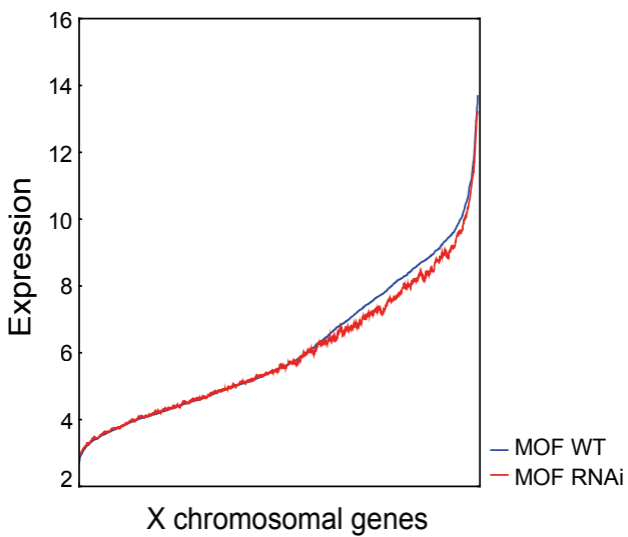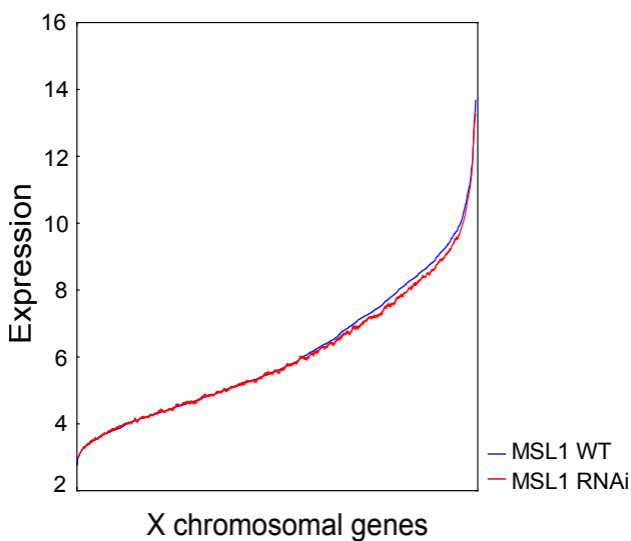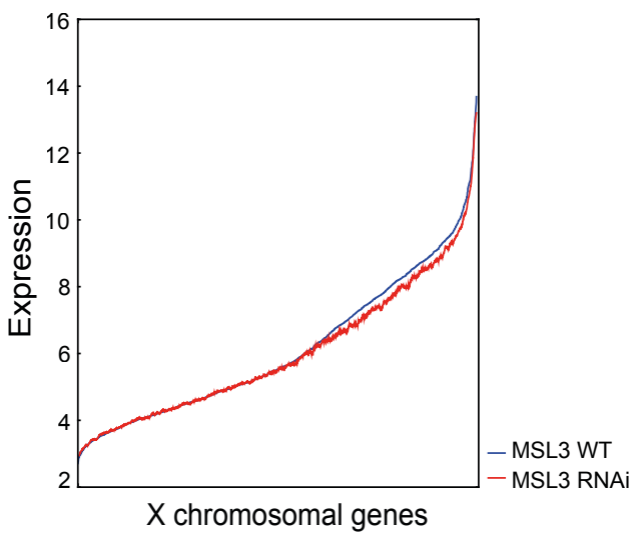

Supplement: Additional file 13 — Expression of X-linked genes in control and in RNAi knockdown of msl-genes. Expression of X-linked genes in EGFP control (blue) and in RNAi knockdown of mof, msl-1 and msl-3 genes (red) in SL-2 cells, sorted by expression in EGFP controls. (A), (B) and (C): line plots of expression in EGFP control and mof RNAi, msl-1 RNAi and msl-3 RNAi cells, respectively. Expression levels in RNAi treated cells are shown as running averages for sets of 21 genes. [file 1471-2164-13-97-S13.PDF]

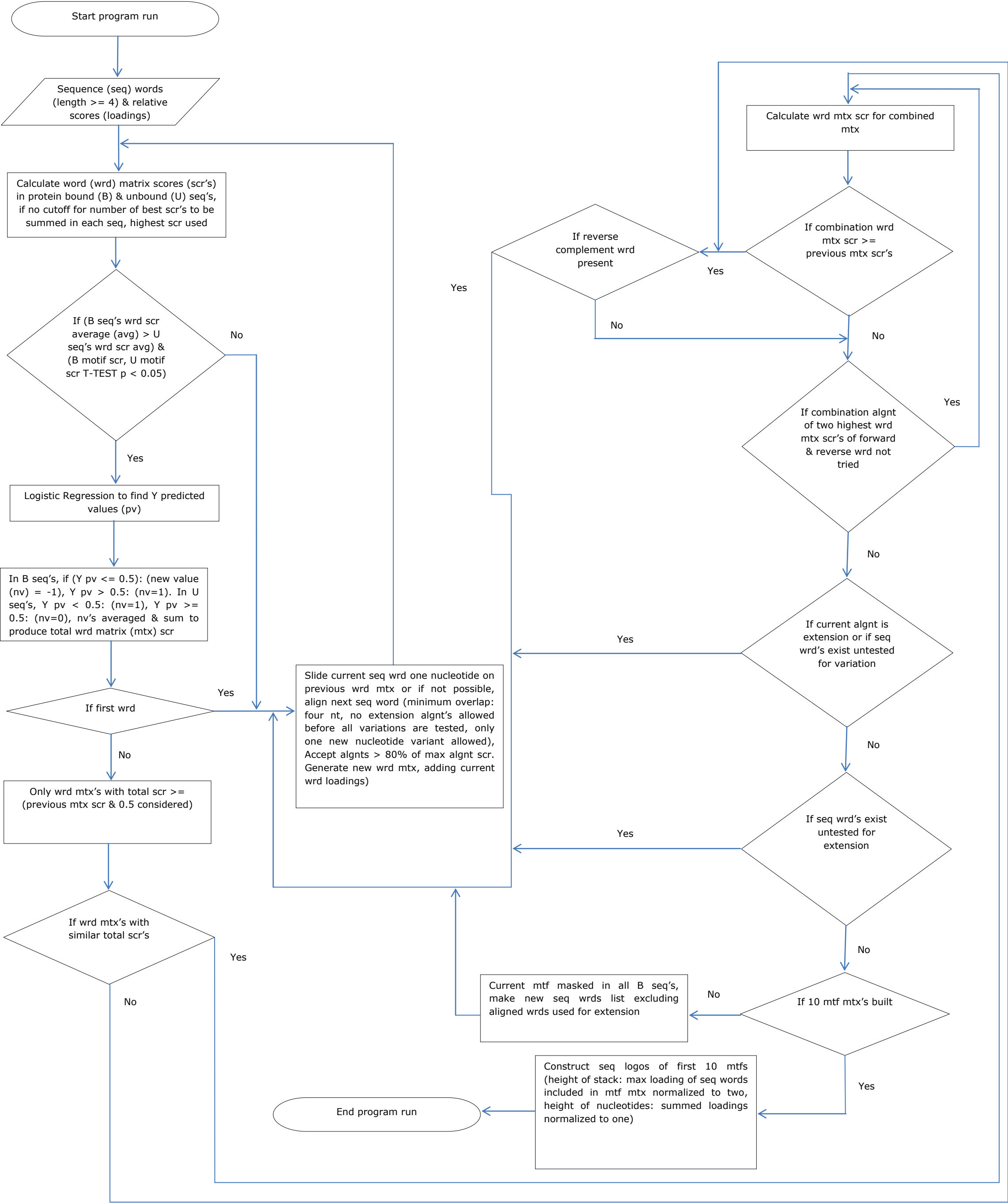

Supplement: Additional file 14 — Sequence word aligner algorithm. A flowchart for the iterative algorithm to identify complex motifs with significant predictive values for protein binding, based on the top sequence words obtained from OPLS-DA models designed to detect sequence differences between genes bound and not bound by the protein. [file 1471-2164-13-97-S14.PDF]
