# Supplementary material for: Sequence signatures involved in targeting the male-specific lethal complex to X-chromosomal genes in Drosophila melanogaster
Source: BMC Genomics. 2012 Mar 19;13:97. doi: 10.1186/1471-2164-13-97 (PMC3355045; doi:10.1186/1471-2164-13-97)
Supplement: Additional file 8 — Correlation between OPLS-DA models and AT-content as well as expression. Results from OPLS-DA models of frequencies of sequence words in features of expressed MSL strongly bound (green, O) vs MSL weakly bound genes (magenta, O) of chromosome X. (A), (B), (C) and (D): scatter plots of first component scores versus AT contents of promoters, coding sequences, introns and 3' UTRs, respectively. (E), (F), G) and (H): scatter plots of promoter, coding sequence, intron and 3' UTR first component scores versus gene expression levels, respectively. [file 1471-2164-13-97-S8.PDF]

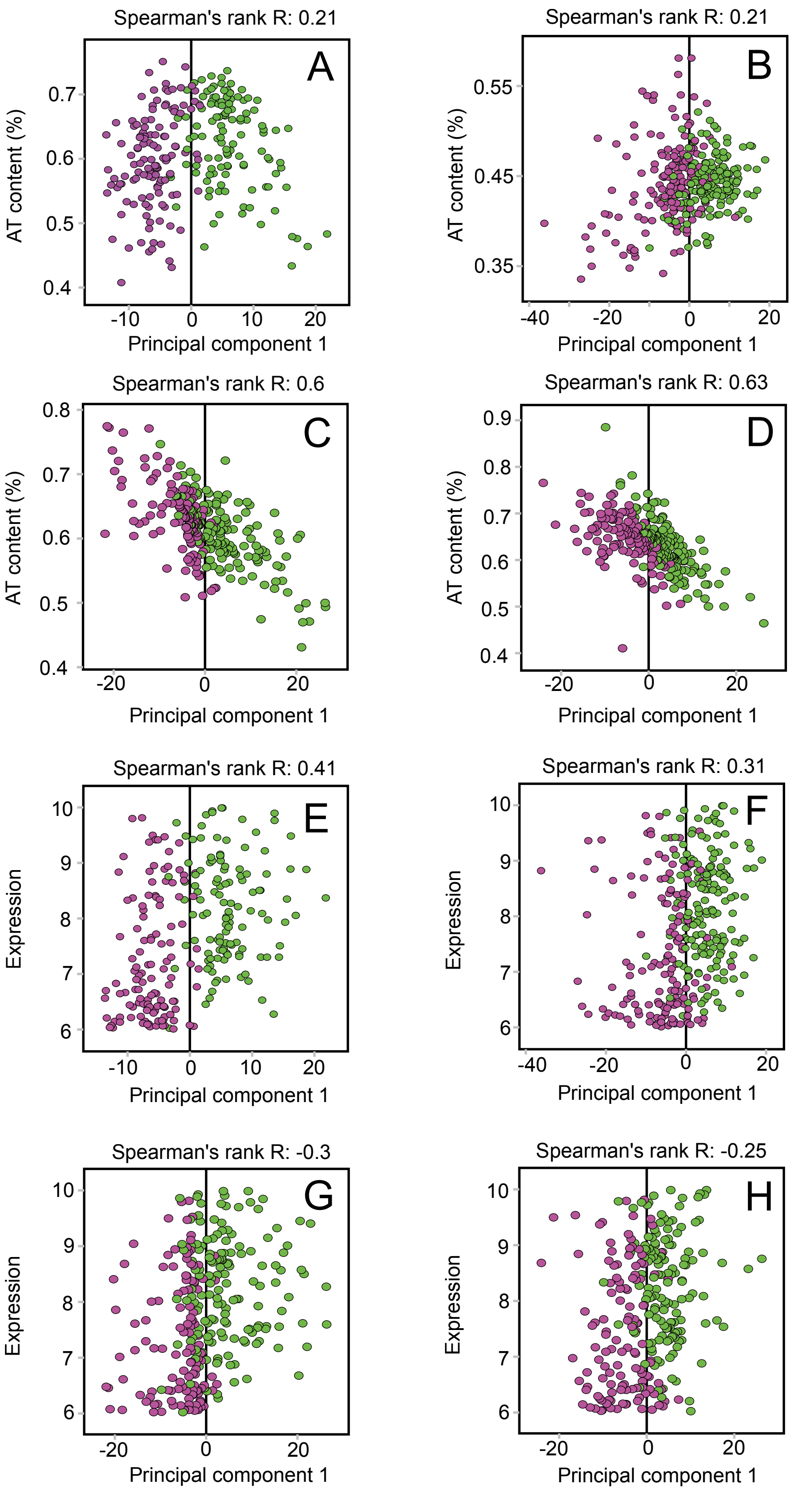

Spearman's rank R: 0.21
A
AT content (%)
Principal component 1
Spearman's rank R: 0.21
B
AT content (%)
Principal component 1
Spearman's rank R: 0.6
C
AT content (%)
Principal component 1
Spearman's rank R: 0.63
D
AT content (%)
Principal component 1
Spearman's rank R: 0.41
E
Expression
Principal component 1
Spearman's rank R: 0.31
F
Expression
Principal component 1
Spearman's rank R: -0.3
G
Expression
Principal component 1
Spearman's rank R: -0.25
H
Expression
Principal component 1
